# Impaired Arithmetic Fact Retrieval in an Adult with Developmental Dyscalculia: Evidence from Behavioral and Functional Brain Imaging Data

**DOI:** 10.3390/brainsci12060735

**Published:** 2022-06-03

**Authors:** Silke M. Göbel, Rebecca Terry, Elise Klein, Mark Hymers, Liane Kaufmann

**Affiliations:** 1 Department of Psychology, University of York, York YO10 5DD, UK; rcterry98@gmail.com; 2 Department of Special Needs Education, University of Oslo, 0371 Oslo, Norway; 3 York Neuroimaging Centre and York Biomedical Research Institute, University of York, York YO10 5DD, UK; mark@hymers.org.uk; 4 LaPsyDÉ, CNRS, Université Paris Cité, 75005 Paris, France; elise.klein@u-paris.fr; 5 Leibniz-Institut fuer Wissensmedien, 72076 Tuebingen, Germany; 6 Department of Psychology, University of Innsbruck, 6020 Innsbruck, Austria; liane.kaufmann@uibk.ac.at

**Keywords:** developmental dyscalculia, arithmetic, fact retrieval, finger counting

## Abstract

Developmental dyscalculia (DD) is a developmental disorder characterized by arithmetic difficulties. Recently, it has been suggested that the neural networks supporting procedure-based calculation (e.g., in subtraction) and left-hemispheric verbal arithmetic fact retrieval (e.g., in multiplication) are partially distinct. Here we compared the neurofunctional correlates of subtraction and multiplication in a 19-year-old student (RM) with DD to 18 age-matched controls. Behaviorally, RM performed significantly worse than controls in multiplication, while subtraction was unaffected. Neurofunctional differences were most pronounced regarding multiplication: RM showed significantly stronger activation than controls not only in left angular gyrus but also in a fronto-parietal network (including left intraparietal sulcus and inferior frontal gyrus) typically activated during procedure-based calculation. Region-of-interest analyses indicated group differences in multiplication only, which, however, did not survive correction for multiple comparisons. Our results are consistent with dissociable and processing-specific, but not operation-specific neurofunctional networks. Procedure-based calculation is not only associated with subtraction but also with (untrained) multiplication facts. Only after rote learning, facts can be retrieved quasi automatically from memory. We suggest that this learning process and the associated shift in activation patterns has not fully occurred in RM, as reflected in her need to resort to procedure-based strategies to solve multiplication facts.

## 1. Introduction

An estimated 5–7% of the general population [1,2,3] suffer from developmental dyscalculia (DD), a specific learning difficulty in acquiring mathematical skills during childhood which persists into adulthood. Poor numeracy in adulthood is more detrimental than poor literacy [4] being associated with lower wages and poor physical [5] and mental [4] health. In comparison to developmental dyslexia, however, the cognitive and neural bases of developmental dyscalculia are currently still under-researched (for a recent review, see [6]). The latest DSM-V diagnostic criteria define DD as a ‘neurodevelopmental disorder of biological origin’ [7]. Very little is known yet about the molecular biological origins of DD, however brain dysfunction has been suggested as a possible biological origin of DD [8]. Despite this only a handful of studies have investigated the neural networks during number and arithmetic processing in adults with DD. The current study investigates the neural networks in an adult with DD during arithmetic processing, compared to a group of typical adults.

### 1.1. Neural Networks for Arithmetic

We start with a review of the literature of neural networks for arithmetic in typical adults. The essential role of the left parietal lobe for arithmetic performance in typical adults has been well-documented for over a century [9]. About 20 years ago, Dehaene proposed more detailed neuroanatomical predictions in his influential model of number processing and arithmetic, the Triple Code Model [10,11]. This model postulates three separate codes for number processing in typical adults: an analogue magnitude code for approximate number processing and magnitude representation, a verbal word frame for number words and verbally-mediated calculation and a visual number form for the processing of Arabic digits. In our study we tightly control for the processing of Arabic digits, thus we will not further discuss research related to the visual number form area here (but for recent neuroimaging research see [12,13,14,15]). 

In a further development of this model, three parietal circuits involved in number processing and arithmetic were highlighted for typical adults [16]. The horizontal parts of the bilateral intraparietal sulci (hIPS) were specified within the intraparietal sulcus (IPS) as the core seat of number semantics, supporting the analogue magnitude representation and expected to be activated in all numerical tasks. The left angular gyrus (AG) was suggested to be involved in the representation of the verbal word frame and number words and to be activated during arithmetic fact retrieval. This was an addition of a parietal area to the previously described fronto-temporal network of perisylvian language areas associated with verbally-mediated arithmetic fact retrieval. A third region in the parietal lobe, the bilateral posterior superior parietal lobules (PSPL), was proposed to support visuospatial representations of number, including the mental number line [16,17], due to its activation in both number comparison and visuospatial tasks [18,19].

Neuroimaging studies over the last 20 years highlighted a large bilateral fronto-parietal network involved in number processing and arithmetic in typical adults [20,21], largely supporting the parietal predictions of the Triple Code Model. This bilateral fronto-parietal network in typical adults has recently been coined the ‘math-responsive network’ [22,23,24]. In particular, the left and right IPS in typical adults have consistently been shown to be activated in number processing [25,26,27] and arithmetic tasks [20,28,29], as well as for quantity representations [30,31]. Studies in typical adults involving number words, exact calculations or training arithmetic facts, on the other hand, which are often learned through verbal rote repetition [32], reported activation of a left-hemispheric network centered around the AG [33,34,35,36,37,38,39]. Furthermore, applying transcranial magnetic stimulation (TMS) over the left but not right AG of typical adults impaired addition performance [40]. Activations in the bilateral PSPL have also been commonly observed during number comparison and arithmetic tasks in typical adults [28,41,42]. However, bilateral PSPL may be involved in orienting attention along the supposedly spatially-oriented mental number line rather than being critical for mental arithmetic itself. While Andres et al. [28] found bilateral PSPL activation in typical adults during both subtraction and multiplication, the application of TMS over PSPL in typical adults affected neither multiplication nor subtraction performance. Overall, the role of the PSPL for number processing and arithmetic in typical adults is less well documented than the involvement of the bilateral IPS and left AG. 

Several studies including meta-analyses [29,30,43,44] augmented the neuroanatomical predictions of the Triple Code Model in typical adults. In the fronto-parietal math-responsive network, those updates further specified frontal brain areas, which are associated with executive functions and working memory [29,43,45]. Additionally, activation of motor cortex and anterior IPS has also been demonstrated in numerical tasks [30,46], especially in those requiring procedural strategies. It has been suggested that this activation in typical adults may reflect finger-counting strategies used in childhood (see [47]; for a detailed behavioral analyses on finger-counting strategies used in an adult with developmental dyscalculia, see [48]). For the encoding of arithmetic facts, there has been increasing evidence in recent years that additional long-term memory processes are involved in typical children and adults, subserved by the medial temporal lobe [21,49]. Thus, Menon [21] proposed that the medial temporal lobe may interact with the angular gyrus to aid long-term memory formation. 

**Subtraction versus multiplication.** So far, we have discussed brain activations related to procedure-based calculation on the one hand and verbal memory-based arithmetic fact retrieval on the other hand, but we have not yet associated these two processing types with specific arithmetic operations (addition, subtraction, multiplication and division). A wealth of behavioral research shows that the processing type used by proficient adults to solve arithmetic tasks is strongly affected by the type of operation [50,51]. For example, typical adults are more likely to solve subtraction than multiplication problems using procedure-based calculation processes, i.e., manipulating the relations between numbers [52], requiring access to the semantic meaning of quantities behind the numbers. In contrast, for multiplication they are more likely to use verbal memory-based arithmetic fact retrieval [53,54]. 

Neuropsychological [55] and neuroimaging [56] studies suggest that those two arithmetic operations might rely on two largely separate neural networks. Studies using cortical electrical stimulation on adult patients undergoing brain surgery have also provided evidence for operation-specificity [57,58,59]. In addition, as described in detail below, the adult with DD who is the focus of the current study, showed significantly stronger difficulties with multiplication than subtraction [48]. Thus, in the current study we aimed to test possible dissociations in the neural networks underlying those arithmetic operations by using a single case design. Previous single case studies of patients with acalculia have contributed to our understanding of arithmetic networks. Lee [60], for example, reported a case study of a patient with acalculia with a lesion including the left angular gyrus. This patient was impaired in multiplication but not in subtraction. Conversely, another patient with a left parietal lesion was impaired in subtraction but not in multiplication [61]. In the Triple Code Model [62] it was suggested that subtraction more than multiplication activates the quantity presentation of numbers in the IPS, while multiplication relies more on verbally mediated fact-retrieval in the left angular gyrus. A stronger involvement of the PSPL in subtraction than multiplication, possibly to move attention along the mental number line was also proposed [16,62]. Several neuroimaging studies support these predictions of partially distinct neural networks for subtraction and multiplication. Greater IPS activation for subtraction over multiplication has been found in many neuroimaging studies with typical adults [54,56,60,63] and some studies [64] also show higher activation in the bilateral PSPL for subtraction than multiplication. Evidence for the left angular gyrus being more important during multiplication than subtraction comes largely from training studies in typical adults [56]. Ischebeck and colleagues [65] demonstrated that increasing familiarity of multiplication problems led to a shift in activity from bilateral IPS to left AG over the course of a single scanning session. While this was initially interpreted as evidence for the involvement of the AG in arithmetic fact retrieval, subsequent studies suggested that the increasing activity in AG did not reflect number-specific fact retrieval but rather a general signature of learning that can be found with various contents [34,66]. Nevertheless, these studies provide evidence that different strategies at the behavioral level (retrieval vs. procedural) are reflected by differential neural activation patterns. Furthermore, TMS to the left AG in typical adults has also led to impaired performance in multiplication but not subtraction [54]. 

However, these distinct networks for subtraction and multiplication have not been found in all studies. Several brain imaging studies observed increased activation in the IPS when solving simple multiplication problems [35,67,68,69,70,71,72]. Rosenberg-Lee et al. [73], for example, reported greater right IPS activation for multiplication than subtraction and TMS over the right IPS in typical adults affected response times for both multiplication and subtraction, but only error rates for multiplication and not subtraction [28]. Other studies using transcranial magnetic stimulation (TMS) also do not consistently show a clear-cut distinction between processing and operation type. For instance, using repetitive navigated transcranial magnetic stimulation with typical adults, Maurer et al. [74] found that multiplication was associated with the left AG, while subtraction revealed the highest association with the right AG, leading the authors to conclude that their data largely confirmed the central assumptions of Triple Code Model. On the other hand, Salillas and colleagues [75] reported an association between multiplication performance and the right intraparietal sulcus by inducing a virtual lesion using transcranial magnetic stimulation in typical adults, pointing to a role of the right hemisphere in multiplication.

In sum, while there is good evidence that the procedures used by typical adults to solve subtraction (mainly calculation) and multiplication (mainly fact retrieval) problems differ, the proposed differential involvement of the IPS has been questioned. One reason for these results could be the flexible use of different strategies by typical adults for both multiplication and subtraction, depending on, for example, task difficulty [54]. Applying rTMS over the hIPS (assumed to be involved in procedure-based calculation) and the left AG (assumed to be involved in verbal memory-based arithmetic fact retrieval) of typical adults, Fresnoza et al. [54] found that the involvement of these brain areas was modulated by the solution strategy employed (i.e., retrieval vs. calculation) rather than by the arithmetic operation per se (i.e., subtraction vs. multiplication). The authors suggested that for typical adults solving less well-established multiplication problems was associated with procedure-based calculation, while solving highly overlearned subtraction problems was associated with verbal memory-based fact retrieval.

Thus, it might be a promising approach to investigate neural networks underlying subtraction and multiplication in adults who use atypical strategies to solve arithmetic problems. One such group are adults with developmental dyscalculia [6]. 

### 1.2. Developmental Dyscalculia

Current diagnostic manuals used for DD, e.g., the DSM-V [7], acknowledge that specific learning disorders tend to persist into adulthood, and thus, should be considered as a lifelong disorder. Developmental dyscalculia manifests itself as severe learning difficulties in mathematics, typically already present during primary school, with mathematical skills well below expected age-level. DD affects functioning at school, work or activities of daily life [6]. Individuals with DD show difficulties in the processing of numerosities [76,77] and in enumeration [78] and struggle with the automatic activation of number magnitude knowledge [79]. Typically, their arithmetic fact retrieval [48,80] and arithmetic conceptual knowledge [81] is impaired.

Several theories about core deficits causing DD have been proposed. Domain-specific theories (e.g., the Approximate Number Sense (ANS) deficit theory [82] and the access-deficit theory [83]) suggest a deficit in the number domain as the root of DD. In the ANS deficit theory DD is related to a less precise approximate number sense that underlies all mathematical problems. The access deficit theory in contrast suggests that the ANS is intact, but that the access from symbolic numbers to the ANS is impaired. Based on reports of non-numerical magnitude processing deficits in DD including conceptual size [84], time/duration [85] and length [86], others [87] have suggested that DD is caused by deficits in a common magnitude system [88] that includes numerical magnitude.

Alternatively, some researchers suggest that the arithmetic impairment in DD reflects domain-general deficits in working memory, visuospatial processing or attention [89]. Working memory is often utilized during arithmetic, for example to temporarily hold the results of a smaller step within a multi-step calculation procedure [90]. Visuospatial processing has been associated with mapping numbers to space in the mental number line [91] which has been suggested as a potential basis for arithmetic learning [92]. Individuals with DD also often show impairments in working memory [93,94] and in visuospatial tasks [95,96].

Finally, it has been suggested that DD can be best described as a disconnection syndrome [97,98,99] in which the primary deficit can be traced to aberrant white matter connectivity between brain regions. Based on diffusion tensor imaging (DTI) data these studies suggest that during ontogenetic development in individuals with DD the frontal lobes become not adequately connected to the parietal lobes which host key structures such as the IPS and AG. Consequently, individuals with DD may struggle with the retrieval of arithmetic facts and accessing non-symbolic magnitude representations even if the representations themselves, and the cortical loci subserving them, are intact. 

Overall, the arithmetic impairment in DD may reflect deficits specific to numerical processing such as magnitude processing or arithmetic fact retrieval, more general cognitive deficits in working memory or visuospatial processing, a combination of these, or a disconnection syndrome between domain-specific and domain-general areas.

**Neural networks activated during arithmetic in DD.** Impaired arithmetic performance in developmental dyscalculia has often been linked to abnormal brain activations in the parietal lobe. However, as far as we are aware, the existing literature on abnormal brain activation during arithmetic in DD is based entirely on studies with children whose arithmetic skills might still be developing. Compared to typically developing children, children with DD [100] showed reduced activation in parietal, visual and prefrontal regions during addition. Parietal under-activation was reported in the right IPS, the right PSPL and the right angular gyrus. Reduced brain activation in children with DD has also been reported during subtraction in the left posterior and inferior parietal lobe [101]. In the same study a classifier was able to distinguish between children with DD and controls based on their activation pattern during subtraction in the angular and inferior frontal gyrus. Finally, reduced activation in children with DD has also been reported for multiplication [102] in the right IPS, SPL and inferior parietal lobe as well as in the left inferior frontal gyrus, and the left middle and superior temporal gyri.

However, at least one study [103] found greater brain activation during subtraction in children with DD than in typically developing children in bilateral IPS, right SPL and prefrontal cortex regions. Moreover, a functional connectivity analysis in the same study demonstrated greater connectivity in children with dyscalculia of the bilateral IPS with the bilateral angular gyri, supramarginal gyri, middle superior and left inferior frontal gyri.

### 1.3. The Current Study 

Most existing knowledge about abnormal brain activations during arithmetic in DD is based on studies of children who are still developing their arithmetic skills. Furthermore, impairments in arithmetic are a key feature of adults with DD. Thus, it is essential to investigate neural networks involved in verbal memory-based arithmetic fact retrieval and procedure-based calculation also in adults with DD. Therefore, the current study aims to contribute to this by investigating neural networks underlying multiplication and subtraction in a single case of a young adult with DD, RM.

RM’s severe arithmetic difficulties, clearly supporting the presence of DD, have been reported elsewhere in detail [48]. In brief, RM used procedure-based calculation strategies more often than memory-based arithmetic fact retrieval not only for subtraction, but also for addition and multiplication. Almost all procedural strategies included counting on her fingers which is highly unusual for an adult. Her arithmetic performance was extremely slow, but surprisingly accurate. RM’s complex use of finger counting methods demonstrated good conceptual and procedural mathematical knowledge. In the current study we compared RMs brain activations during multiplication and subtraction to those of a control group of typical adults.

**Hypotheses.** First, for the control group we expected stronger activation in the typical bilateral fronto-parietal math-responsive network of procedure-based calculation during subtraction and in the left lateralized arithmetic fact retrieval network of perisylvian language areas centered around the left AG during multiplication. Furthermore, we predicted stronger activations in bilateral IPS and PSPL for subtraction than multiplication and stronger activation in the left AG during multiplication than subtraction. 

Second, and most importantly, we expected RM’s patterns of brain activation during arithmetic to differ significantly from controls. We predicted that RM would activate the math-responsive network during both multiplication and subtraction. Given her reduced use of arithmetic fact retrieval, we predicted that she would show less brain activation for multiplication in the left AG. We also expected her to rely more on accessing representations of magnitude and relations between numbers. Therefore, we predicted higher activations in the IPS and PSPL, bilaterally, during both multiplication and subtraction. Given RM’s extensive use of finger counting we also expected higher activation of the motor cortices for RM during both multiplication and subtraction. 

## 2. Materials and Methods

### 2.1. Participants

All participants had normal or corrected to normal vision and no history of neurological disorders. Participants were students of Psychology or Cognitive Neuroscience at the University of York, U.K., provided written informed consent and were reimbursed for their time. The study was approved by the Ethics and Sciences Committees of the York Neuroimaging Centre (YNIC), University of York.

#### 2.1.1. Single Case 

RM was a left-handed, 19-year-old, female, second-year undergraduate Psychology student at the University of York who self-reported long-lasting difficulties with arithmetic since childhood. RM’s behavioral and neuroimaging data were collected over a period of six months in 2009/2010. As reported elsewhere in detail (see [48]), RM had severe difficulties in arithmetic despite having average general cognitive skills, average reading and spelling skills and average working memory (WM) resources (see Table 1). Her performance on a standardized test of arithmetic (WRAT-3 arithmetic [104]) was below average (SS of 80). Her performance of oral arithmetic with single-digit operands (for a detailed report see [48]) was highly accurate, very slow and atypical for an adult. Apart from rule-based facts (i.e., n × 1, n × 0) and the 5-times table, she very rarely used fact retrieval. For instance, she did not even use her 2-times table knowledge consistently. Indeed, procedural strategies were used considerably more than fact retrieval, even for simple facts such as 4 × 3. She used finger counting for almost every calculation. For subtraction problems the most frequently used strategy was ‘counting up/down by one’, for multiplication problems ‘counting up by ones and twos’ and a combination of ‘retrieval of a 5-table with subsequent counting up/down’.

#### 2.1.2. Control Group

We recruited an opportunity sample of 18 right-handed control participants aged 19 to 37 years (mean age 22.06 years, SD = 4.45, 10 female) without a diagnosis of developmental learning difficulties (including developmental dyslexia and dyscalculia) and with at least average performance on a standardized test of arithmetic (mean standard score of 100.33, SD = 10.17) and at least average general cognitive, reading and spelling skills (see Table 1).

### 2.2. Stimuli

#### 2.2.1. Background Measures

RM was assessed on a range of cognitive background measures. All control participants were tested on the same background measures, apart from the working memory measures. Due to time restrictions, we were able to test working memory only for 10 of the control participants. 

**General cognitive ability.** The cognitive abilities of the participants were estimated using the Wechsler Abbreviated Scale of Intelligence (WASI; [109]). A total of 16 participants (including RM) were tested on all four subtests (Vocabulary, Block Design, Similarities, Matrix Reasoning); for 3 control participants, we estimated their cognitive ability based on their performance on two subtests (Vocabulary, Matrix Reasoning).

**Arithmetic.** Arithmetic ability was assessed using the Wide Range Achievement Test (WRAT-3 Arithmetic [104]) arithmetic subtest. Participants were given 15 min to work through the 40 mathematical problems of increasing difficulty (from additions, subtractions, multiplications, divisions, fractions, to the use of decimal numbers, and algebra). 

**Reading skills.** The participants’ ability to pronounce printed words accurately and fluently was assessed with the word (Sight Word Efficiency) and nonword (Phonemic Decoding) subtests of the Test of Word Reading Efficiency (TOWRE; [110]). Reading and spelling accuracy were established by administering the Reading and Spelling Subtests of the Wide Range Achievement Test (WRAT–3 Reading, WRAT–3 Spelling; [104]).

**Working memory.** We tested RM and 10 control participants on two tests from the Wechsler Memory Scale (WMS–III; [111]): Digit Span Forward and Digit Span Backward. In Digit Span Forward, participants heard sequences of numbers increasing in length and had to repeat them verbatim. During Digit Span Backward they also heard sequences of digits but had to repeat them in reverse order. In both tests, sequences increased in length until the participant recited two sequences of the same length incorrectly, at which point the testing finished. To assess nonverbal memory skills, participants were tested on the Spatial Span Forward and Spatial Span Backward subtests of the Wechsler Memory Scale (WMS–III), which are spatial analogues of the Digit Span tests. A board that had nine 3-dimensional cubes placed on it was shown to the participants. The experimenter pointed to a sequence of cubes one after the other. The participant had to remember the sequence and repeat it either in the order presented (Spatial Span Forward) or in reverse order (Spatial Span Backward). Sequences increased in length until participants produced two sequences of the same length incorrectly.

**Handedness.** Participants’ handedness was assessed with the Edinburgh Handedness Inventory [112].

#### 2.2.2. fMRI Tasks

**Figure 1:**
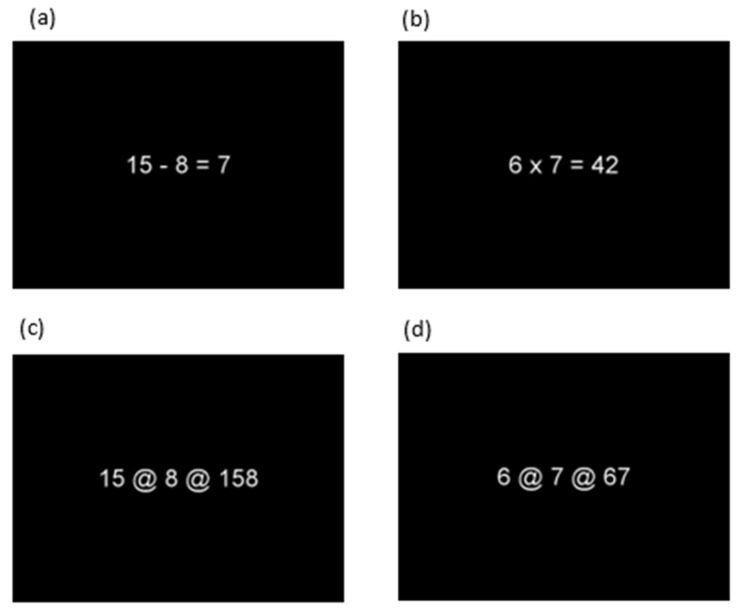
Examples of (**a**) a subtraction trial, (**b**) a multiplication trial, with the corresponding control trials (panels (**c**,**d**)).

In the fMRI scanner, participants were shown subtraction, multiplication, and control problems with two operands and a suggested solution (presented centrally as white text on a black background, Arial, font size = 30 points). Examples of different trial types are shown in Figure 1. A list of all items is provided in Table A1 in Appendix A. Each problem was presented for 3 s, and the inter-trial interval (ITI) varied between 0 and 12.5 s (mean ITI = 2.8 s). Fifty percent of the suggested solutions were incorrect. Participants had to indicate whether the solution presented was correct or incorrect by pressing a button with their right (correct) or left (incorrect) index finger as quickly and accurately as possible.

**Subtraction:** Half of the subtraction trials were problems with a small problem size, i.e., the first operand was a double digit ranging from 11 to 19, and the second operand was a single digit ranging from 3 to 9. The other half had a large problem size, i.e., the first operand ranged from 52 to 91 and the second from 23 to 76. Overall, 80 subtraction trials were presented.

**Multiplication:** All multiplication items (n = 80) used only single-digit operands from 2 to 9. Half of the incorrect solutions were table-related (e.g., the incorrect answer is from the same times table, e.g., 2 × 8 = 14) and half were table-unrelated (e.g., 2 × 8 = 17).

**Control:** For both the subtraction and multiplication task, each problem had a corresponding control item (see Figure 1, panels c and d) which showed the same operands with the arithmetic operational signs replaced by a non-numerical symbol (@). The participants had to judge whether the digits after the second @ were in the same order as the digits presented before. Correct control items (50%) showed the operands after the second @ in the same correct order (e.g., 15 @ 8 @ 158), incorrect control items (50%) in the reversed order (e.g., 15 @ 8 @ 815). These control items were chosen to control for supporting task components (e.g., reading, number processing, decision making and manual responses) without performing an arithmetic operation.

#### 2.2.3. MRI/fMRI Acquisition

MRI imaging was conducted on a GE 3T Excite HDx scanner (GE Healthcare) using an eight-channel phased-array head coil (GE Signa Excite 3.0T, High-Resolution Brain Array, MRI Devices Corp., Gainesville, FL). Head movement was minimized using foam inserts. Stimuli were projected onto a screen and viewed by participants using a mirror attached to the head coil.

Functional MR imaging collected 38 sagittal slices per volume with no inter-slice spacing using a T2*-weighted gradient echo EPI sequence (TR = 3 s, TE = 34.3 ms, field of view (FOV) = 256 mm, matrix size = 128 × 128, voxel size = 2 × 2 × 3.5 mm^3^, slice thickness = 3.5 mm, flip angle = 90°).

High resolution anatomical images were acquired using a T1-weighted sagittal isotropic 3D FSPGR sequence (TR = 9 ms, TE = 3.6 ms, FOV = 256 mm, matrix size = 256 × 256, voxel size = 1 × 1 × 1 mm^3^, slice thickness = 1 mm, flip angle = 8°). For two control participants, the voxel size was 1 × 1.13 × 1.13 mm^3^.

### 2.3. Procedure

Participants were tested in two testing sessions. In the first testing session the background cognitive tests were administered in a quiet room in the Department of Psychology at the University of York.

The second testing session took place on a separate day at the York Neuroimaging Centre. Before entering the scanner, participants were given a practice run of all tasks and trial types; they were asked to judge stimuli presented to them on a computer screen and to respond by pressing one of two buttons as accurately and quickly as possible. 

Each participant then took part in two scanning sessions on the same day with a short break between the sessions. The order of the scanning sessions was counterbalanced between participants. Each scanning session began with a 6-min localizer block unrelated to the current study. The localizer block was followed by two multiplication and two subtraction blocks. Multiplication and subtraction blocks alternated with one scanning session starting with a multiplication and the other with a subtraction block. Each calculation block lasted four minutes. An additional two volumes (6 s) were acquired before the start of each block, resulting in a total of 22.5 min per scanning session. Instructions for each task were presented before each block. Response times and accuracy were recorded.

For each calculation block an event-related design was used. In each subtraction and multiplication block 40 items were presented with 20 calculation and the 20 corresponding control items intermixed pseudo-randomly (multiplication block: 20 multiplication items, 20 multiplication control items; subtraction block: 20 subtraction items, 20 subtraction control items). Stimulus timing and inter-trial interval (ITI) duration within blocks were optimized and randomized using AFNI software [113].

Finally, an anatomic T1 scan was obtained unless the participant already had structural data from previous studies at the York Neuroimaging Centre.

### 2.4. Data Analyses

#### 2.4.1. Behavioral Analyses

RM’s performance on the background measures and her accuracy and responses times in the fMRI tasks were compared to the performance of the control group using a modified *t*-test. The test was developed by Crawford and Howell [105] to compare a single subject to a sample of control participants. In this method, the individual is treated as a sample of N = 1, and a modified version of an independent samples *t*-test is used to compare the individual to a normative sample. The *t*-tests were implemented using the Singlims_ES.exe program [106,114]. The Benjamini-Hochberg [107] method of controlling the false discovery rate (FDR) was used to correct for multiple comparisons, which increase the risk of type I errors. We chose to apply the Benjamini-Hochberg correction because it is better able to reduce false positives while minimizing false negatives than more conservative measures [108]. Benjamini-Hochberg adjusted *p*-values were calculated using the method described by Jafari and Ansari-Pour [108]. 

#### 2.4.2. fMRI Analysis

**Data pre-processing.** All fMRI data were analyzed using FEAT (FMRI Expert Analysis Tool, Version 6.00) part of FSL (FMRIB’s Software Library [115]). The Brain Extraction Tool (BET [116]) was used to extract the brain from EPI data, and a temporal high pass filter of 0.02 Hz was applied. The images were motion-corrected using MCFLIRT [117], slice timing corrected, and a Gaussian smoothing kernel of FWHM 5 mm was applied. The functional images were registered to the individual’s structural T1 scans using FLIRT [117,118] and were co-registered to a standard 2 mm MNI152 brain using FNIRT nonlinear registration [119].

**Single-subject analysis.** Individual participants’ whole-brain responses were modeled using a general linear model with each experimental condition (e.g., multiplication trials, multiplication controls trials) used as a regressor. The regressors were defined as the trial duration (3000 ms) convolved with FSL’s canonical gamma hemodynamic response function. Only trials on which participants responded correctly were included in the analysis. Our data analyses focused only on items which presented correct solutions and on the following contrasts of parameter estimates (COPEs): multiplication > multiplication control, subtraction > subtraction control, multiplication minus multiplication control > subtraction minus subtraction control (for simplicity called multiplication > subtraction) and vice versa (for simplicity called subtraction > multiplication). The two scanning sessions were first analyzed separately for each participant and then combined separately for each participant by using a fixed-effect analysis, thresholded at *p* < 0.05.

**High-level whole-brain analysis.** For each of the contrasts, the COPE files created from the combined subtraction/multiplication analyses for all control group participants were combined to calculate a group mean activation using a mixed effect analysis with FLAME [120]. The statistical maps were corrected for multiple comparisons using a cluster threshold method with a Z threshold of 2.3 and a cluster *p* threshold of *p* < 0.05. Due to large clusters being identified, the analyses of subtraction > control and subtraction > multiplication were re-run with a Z threshold of 3.1. To investigate differences between the single case RM and the control group, a second FLAME analysis [120] was run, including RM as a separate group for each contrast of the original analysis. This identified areas of higher activation for the control group than RM and areas of higher activation for RM compared to the control group in the multiplication and subtraction tasks. These statistical maps were also corrected for multiple comparisons and used the same threshold as the control group mean analysis (*Z* = 2.3 and cluster *p* < 0.05).

**Regions of interest (ROI) analysis.** In the Regions of Interest (ROI) analysis we focused on brain regions for which we had clear predictions of significant differences in brain activation between RM and the control group. These areas were four areas in each hemisphere: the intraparietal sulcus (IPS), the angular gyrus (AG), the posterior superior parietal lobule (PSPL) and the primary motor cortex. These areas were defined using the Jülich Histological Atlas [121,122,123]. The Jülich Atlas splits these regions into further anatomical subregions based on probabilistic cytoarchtectonic maps: the IPS into hIP1, hIP2 and hIP3 [124,125], the PSPL into 7A, 7M, 7PC and 7P [125], the angular gyrus into PGa and PGp [126] and the motor cortex into 4a and 4p [127]. For our ROI analyses we calculated the mean percentage BOLD signal change for each subregion and then averaged across the subregions.

For each participant, separate contrasts were run to calculate differences in BOLD signal change for the multiplication and subtraction trials showing correct solutions against baseline activation. The resulting COPE files were used in the ROI analysis. FEAT Query was used to extract mean percentage signal change.

To investigate differences in mean signal change between multiplication and subtraction in the control group ANOVAs were run using IBM SPSS version 27. First, a 2 × 2 (task × hemisphere) repeated measures ANOVA was used to compare mean percentage signal change in the IPS for task (multiplication vs. subtraction) and hemisphere (left vs. right). A one-way repeated measures ANOVA was used to compare mean percentage signal change in multiplication vs subtraction for the left angular gyrus. An additional 2 × 2 repeated measures ANOVA was run to compare differences in mean signal change for task (multiplication vs. subtraction) and hemisphere (left vs. right) in the PSPL.

Modified *t*-tests [105] were used to compare the mean percentage signal change for RM separately for each region to the mean percentage signal change of the control group and the Benjamini-Hochberg correction [107] was applied to correct for multiple comparisons. 

## 3. Results

### 3.1. Behavioral Results

#### 3.1.1. Background Measures

Table 1 shows, in line with the selection criteria, that RM performed lower than controls on the WRAT arithmetic test. However, while RM’s arithmetic performance was below average (SS = 80) and the control group performed in the average range, a direct statistical comparison just failed to reach significance (*p* = 0.058). There was no significant difference in performance between RM and the control group on general cognitive skills, reading performance and working memory measures with the exception of the Spatial Span Forwards test. RM’s performance on the Spatial Span Forwards (Scaled Score = 7), while in the average range, was significantly lower than for the control group. Similarly, while RM’s performance on the WRAT spelling test was average, it was significantly lower than for the control groups. None of these differences stayed significant when controlling for multiple comparisons. 

#### 3.1.2. Behavioral Results of the Calculation Tasks Performed in the fMRI Scanner

Table 2 shows the accuracy and reaction times (RT) for RM and the control group for the calculation and control tasks performed in the fMRI scanner. 

**Control group only.** Two repeated-measures analyses of variance (ANOVAs) with item type (multiplication, subtraction) and task (calculation, control) as within-subject factors were conducted on mean accuracy and mean RTs of correct responses. Overall, control participants were significantly more accurate on control than calculation items (*F* (1.17) = 41.758, *p* < 0.001, *η_p_*^2^ = 0.711) and more accurate on multiplication than subtraction items (*F* (1.17) = 34.571, *p* < 0.001, *η_p_*^2^ = 0.670). The interaction was also significant (*F* (1.17) = 36.150, *p* < 0.001, *η_p_*^2^ = 0.680), indicating that accuracy on the subtraction calculation items was particularly low. As expected, response times were significantly faster on control than calculation items (*F* (1.17) = 113.301, *p* < 0.001, *η_p_*^2^ = 0.870). Control participants took significantly longer to respond to subtraction than to multiplication items (*F* (1.17) = 147.480, *p* < 0.001, *η_p_*^2^ = 0.897). The interaction was also significant (*F* (1.17) = 66.112, *p* < 0.001, *η_p_*^2^ = 0.795), indicating that response times on the subtraction calculation items were particularly slow.

**Comparison between RM and control group.** RM’s accuracy was significantly lower than the average accuracy of the control group for both types of calculation trials (multiplication and subtraction). However, after correcting for multiple comparisons, this difference remained significant only for multiplication calculation trials. RM’s accuracy on both types of control trials (multiplication control, subtraction control) was not significantly different from the control group. While RM’s response times were longer on all trial types, only RM’s response times on the multiplication calculation trials were significantly lower than those of the control group. This difference remained significant after correcting for multiple comparisons.

### 3.2. fMRI Results

#### 3.2.1. Whole-Brain Analysis

##### Control Group Only

**Figure 2:**
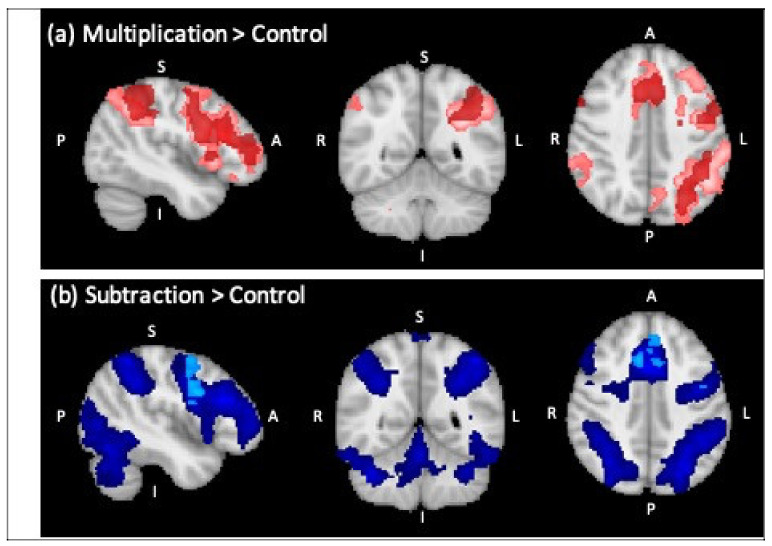
Overlay of regions of significant BOLD signal change for the control group in dark color and for RM in light color (sagittal, coronal and axial view from left to right). (**a**) Multiplication versus control (cluster threshold Z = 2.3) (RM in light red, control group in dark red), (**b**) subtraction versus control (cluster threshold Z = 2.3) (RM in light blue, control group in dark blue).

In the control group, comparing multiplication (with correct solutions) to control trials revealed a largely left-lateralized parietal-frontal network comprising left IPS, AG, supramarginal gyrus and PSPL (Table 3, Figure 2). Frontal clusters of activation included the left inferior frontal gyrus, left and right middle frontal gyri, and the paracingulate gyrus. 

Comparing subtraction (with correct solutions) to control trials yielded one large parieto-frontal cluster of activated voxels at the initial threshold of Z = 2.3. At a more conservative threshold of Z = 3.1, three bilateral fronto-parietal clusters of activation remained significant (Table 3), covering the typical bilateral fronto-parietal math-responsive network (e.g., Amalric & Dehaene, 2017). In particular, the frontal cluster centered around left inferior frontal gyrus extended into the frontal poles of both hemispheres as well as the bilateral middle and inferior frontal gyri. Bilateral parietal clusters included the IPS, superior parietal lobule, AG and supramarginal gyrus.

**Figure A1:**
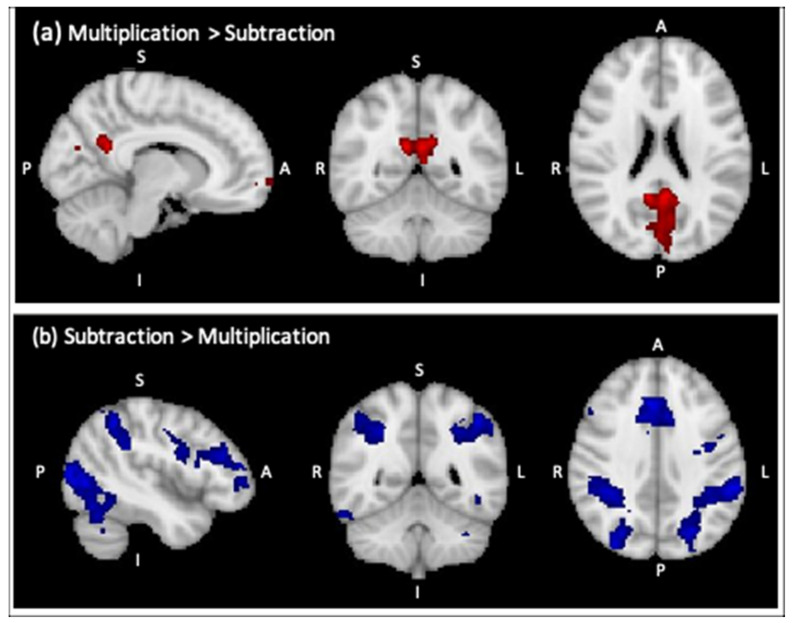
Regions of significant BOLD signal change for the control group (sagittal, coronal and axial view from left to right). (**a**) multiplication > subtraction (cluster threshold Z = 2.3), (**b**) subtraction > multiplication (cluster threshold Z = 3.1).

There were only two small clusters of activation showing higher activation during multiplication calculation than subtraction calculation (Table 3). As can be seen in Figure A1a in Appendix B there was a medial cluster that extended from the visual cortex to the posterior cingulate gyrus and included the left precuneus cortex. There was also a small cluster of activation in the left frontal pole. 

For the identification of brain areas with higher activation for subtraction calculation trials than multiplication calculation trials, using a threshold of 2.3 one large cluster emerged, so a higher threshold of Z = 3.1 was applied. As can been seen in Figure A1b in Appendix B, using a higher threshold of Z = 3.1 showed the typical math-responsive network (bilateral clusters of activation in occipital, parietal and frontal areas). The bilateral parietal clusters included the IPS, superior parietal lobule, AG and supramarginal gyrus, and the frontal clusters included the left precentral and middle frontal gyrus, the right middle frontal gyrus and anterior cingulate gyrus. 

##### Comparison between RM and the Control Group

**Figure 3:**
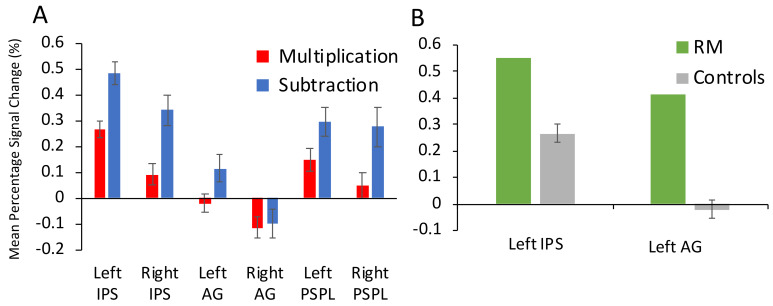
(**A**) Mean percentage signal change in six ROIs (left and right IPS, left and right AG, and left and right posterior superior parietal lobule) by task (multiplication in red, subtraction in blue) for the control group, (**B**) Mean percentage signal change during multiplication in the left IPS and the left AG for RM (in green) and the control group (in gray).

Whole-brain comparison of brain activation in RM to the control group revealed clusters of higher activation for RM than the control group for multiplication versus control (see Table 4, Figure 3a). However, we failed to find any significant clusters of higher brain activation for RM than the control group for subtraction versus control. 

**Figure 4:**
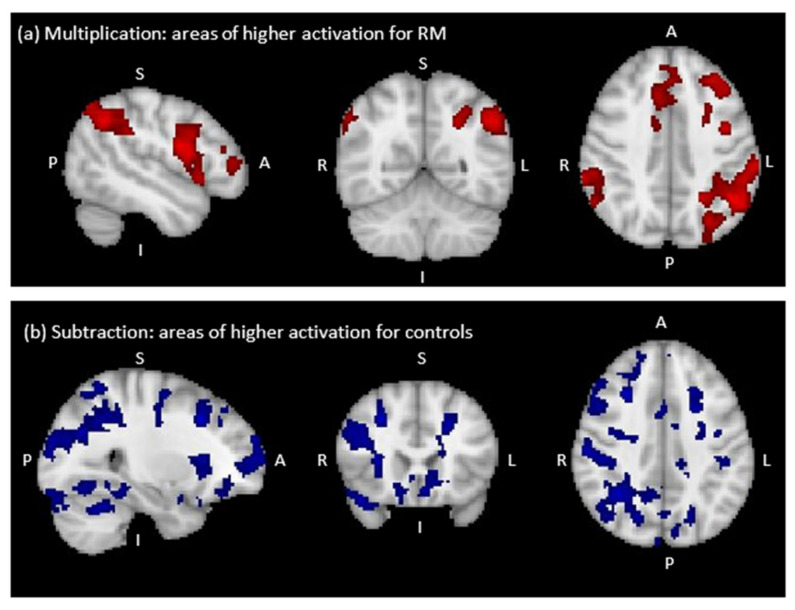
Regions of significant differences in BOLD signal change between RM and the control group (sagittal, coronal and axial view from left to right). (**a**) Higher activation for RM than the control group in multiplication versus control (cluster threshold Z = 2.3), (**b**) Lower activation for RM than the control group in subtraction versus control (cluster threshold Z = 3.1).

As can been seen in Figure 4a, brain areas with significantly higher activation during multiplication in RM than in the control group included the left middle and inferior frontal gyri and a large left parietal cluster covering the IPS, AG, supramarginal gyrus and superior parietal lobule. There was also a small cluster of activation located near the right supramarginal gyrus. There were no brain regions that showed higher activation for the control group than for RM during multiplication.

We failed to find any significant clusters of higher brain activation for RM than for the control group during subtraction. However, as can be seen in Figure 4b the higher brain activation in the control group than in RM during subtraction was largely right-lateralized, comprising right frontal pole, middle frontal gyrus, IPS, supramarginal gyrus and superior parietal lobule. Small clusters were also found in the left supramarginal gyrus and superior parietal lobule.

##### 3.2.2. Regions-of-Interest Analysis (ROI)

The mean percentage signal change by ROI (the left and right IPS, left and right AG, left and right PSPL and left and right motor cortex (motor)) for multiplication and subtraction can be seen in Table 5 separately for RM and for the control group. 

##### Control Group Only

Figure 3A shows the mean percentage signal change for the control group by task for each of the parietal ROIs. When mean percentage signal change in the left and right IPS was compared across tasks (multiplication, subtraction) with a 2 × 2 repeated measures ANOVA a significant main effect of task (*F* (1.17) = 26.93, *p* < 0.001, *η*_p_^2^ = 0.613) and of hemisphere (*F* (1.17) = 34.25, *p* < 0.001, *η*_p_^2^ = 0.668) emerged. There was no significant interaction between task and hemisphere (*F* (1.17) = 1.297, *p* = 0.271, *η*_p_^2^ = 0.071). As can be seen in Table 5, mean percentage signal change was significantly higher for subtraction (left IPS: 0.48% and right IPS: 0.34%) than multiplication (left IPS: 0.27%, right IPS: 0.09%) and mean percentage signal change was higher in the left than in the right IPS for both tasks. 

In the left AG, a one-way repeated measures ANOVA showed a significant effect of task on mean percentage signal change (*F* (1.17) = 7.038, *p* = 0.017, η_p_^2^ = 0.293). The percentage signal change in the left AG was significantly higher for subtraction (0.12%) than for multiplication (−0.02%).

When mean percentage signal change in the left and right PSPL was compared across tasks (multiplication, subtraction) with a 2 × 2 repeated measures ANOVA, there was a significant main effect of task (*F* (1.17) = 7.718, *p* = 0.013, *η*_p_^2^ = 0.312) but no significant effect of hemisphere (*F* (1.17) = 2.865, *p* = 0.109, *η*_p_^2^ = 0.144). Mean percentage signal change in PSPL was higher during subtraction than multiplication. The interaction between task and hemisphere was also significant (*F* (1.17) = 5.674, *p* = 0.029, *η*_p_^2^ = 0.250). The mean percentage signal change during multiplication was smaller in the right PSPL (0.05%) than in the left PSPL in multiplication (0.15%).

##### Comparison between RM and the Control Group

Descriptively, RM showed a higher mean percentage signal change than controls in all ROIs during multiplication (Table 5). However, statistically significant differences were only found for the left IPS and left AG (see Figure 3B). There was a significantly larger increase in BOLD signal in the left IPS for RM than the control group during multiplication (0.55% compared to 0.27% [*t*(17) = 1.88, *p* = 0.039, one-tailed]). Similarly, RM showed a mean percentage signal change of 0.41% in the left AG, which was significantly higher than the control group mean of −0.02% during multiplication [*t*(17) = 2.72, *p* = 0.007, one-tailed]. However, these differences in percentage signal change for both ROIs were no longer significant once corrected for multiple comparisons (Table 5). Interestingly, before correction for multiple comparisons, the difference in percentage signal change during multiplication between RM and the control group was marginally significant for both left and right motor cortex, with higher signal change for RM.

While descriptively RM showed a lower mean percentage signal change than controls in all ROIs during subtraction, no regions showed a significant difference between RM and controls in the subtraction task (Table 5). However, before correction for multiple comparisons, the difference in percentage signal change between RM and the control group during subtraction was marginally significant for both left and right IPS, for left PSPL and for the right motor cortex, with lower signal change for RM.

## 4. Discussion

The aim of this study was to investigate differences in brain activation during arithmetic in an adult with developmental dyscalculia (DD) compared to a typical control group. We tested participants on two arithmetic operations, multiplication and subtraction. Previous research indicated that typical adults use mainly verbal memory-based processing during multiplication and more frequently procedure-based calculation during subtraction [51,53,132,133,134,135]. Those processing types rely on two different brain networks, which interact but are anatomically largely separate [49,136]. 

We only partially replicated the previously described dissociation in brain activation for multiplication and subtraction. Controls activated bilateral frontal and left parietal regions, including left IPS and AG, during both multiplication and subtraction. In line with our predictions, the mean percentage signal change in the bilateral IPS and PSPL was significantly higher for subtraction than for multiplication. However, in contrast to our hypothesis, controls also showed a significantly greater signal change for subtraction than multiplication in the left AG.

Compared to the controls, RM, the adult with DD, showed atypical brain activation patterns during both operations. As predicted, during subtraction, RM showed lower activation in the IPS and the PSPL than the controls and during multiplication RM showed higher left IPS activation than the controls. RM also showed a tendency to activate the left and right motor cortices more strongly than controls during multiplication. Contrary to our expectations, RM had significantly higher activations than controls during multiplication in the left AG and right supramarginal gyrus and RM did not show higher motor cortex activation than controls during subtraction. 

### 4.1. Arithmetic Networks in Typical Adults during Subtraction and Multiplication

Our results of brain activations in typical adults during arithmetic contribute to a large evidence base highlighting the involvement of the IPS in mental arithmetic [20,29,30,63,137]. We strictly controlled for number processing (e.g., identification of Arabic digits, magnitude activation) by using a closely matched control task, so our results highlight that the involvement of the IPS to arithmetic goes beyond just processing symbolic numbers. 

In line with the previous literature, we found a largely left-lateralized network including the AG during multiplication [34,65,67,138], while subtraction was associated with a bilateral activation pattern including both left and right IPS [54,60,63]. However, regarding the left IPS and left AG this distinction was not as clear-cut as proposed in the literature. During multiplication the left IPS was also activated, and during subtraction we also found significant activation in the left AG. 

In line with the literature suggesting stronger IPS activation for subtraction, in our study the signal change in the left IPS was higher during subtraction than during multiplication. Furthermore, as expected AG activation during subtraction was significantly weaker than IPS activation during subtraction. However, it has to be noted that subtraction was in general relatively more difficult than multiplication for the control groups, as can be seen from significantly slower reaction times and higher error rates in behavioral data. In line with this, the fMRI signal change was also stronger in subtraction than in multiplication not only in left IPS but in all ROIs (Figure 3A). This finding is consistent with more effort and more activation for more difficult tasks. Thus, due to this difficulty effect direct comparisons of activation levels between the two operations of multiplication and subtraction should be viewed with caution for the control participants. However, we wish to note that the difficulty effect in controls was a secondary and necessary effect of the case-control design of this study: due to RM’s multiplication deficit, we had to use simple multiplication items so that she was able to perform the task. This means that multiplication and subtraction were thus matched by difficulty for RM but not for the control group. 

**Figure 5:**
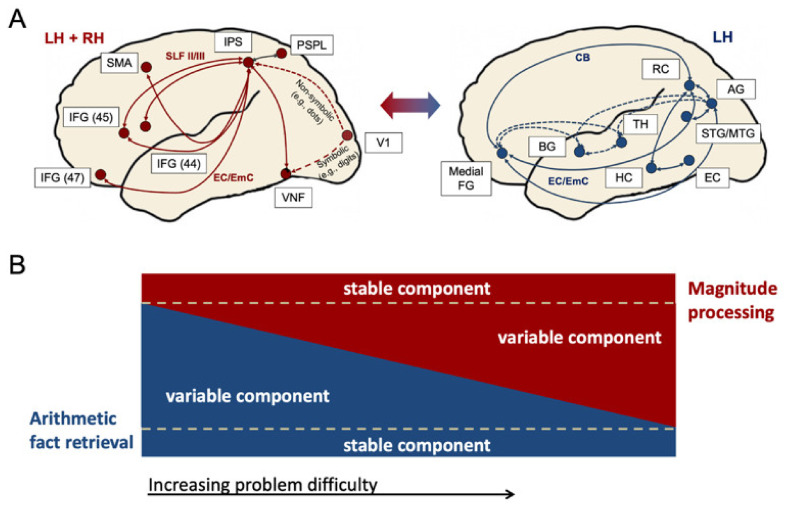
(**A**) Cortical networks and processing pathways for magnitude processing (left panel, red) and arithmetic fact retrieval (right panel, dark blue) in adults with and without DD (developed based on [6,49]). The color-changing arrow between the two panels reflects that these two anatomically separate networks operate together as functionally integrated circuits in numerical cognition. (**B**) Interplay between magnitude processing and arithmetic fact retrieval. A certain amount of fact retrieval is assumed to be involved in arithmetic independent of the difficulty of the task, depicted as the blue rectangle at the bottom. However, the harder a task becomes, the less fact retrieval (blue triangle, variable fact retrieval component) and the more magnitude processing has been assumed (red triangle, variable magnitude processing component). On the other side, an invariant component of mandatorily involved magnitude processing is assumed as well, depicted as the red rectangle at the top of the figure. *Abbreviations:* AG—angular gyrus; BG—basal ganglia; EC—entorhinal cortex; EC/EmC—external/extreme capsule system; HC—hippocampus; IFG—inferior frontal gyrus; IPS—intraparietal sulcus; LH—left hemisphere; Medial FG—medial frontal gyrus; MTG—middle temporal gyrus; PSPL—posterior superior parietal lobule; SLF—superior longitudinal fascicle; SMA—supplementary motor area; RC—retrospenial cortex; RH—right hemisphere; TH—thalamus; V1—primary visual cortex; VNF—visual number form.

What can be noted, however, is that in controls (1) left IPS activation was found during multiplication and (2) left AG activation during subtraction. This observation can be explained by the following version of the Triple Code Model [49]: According to Klein et al. the cortical networks and processing pathways for magnitude processing and arithmetic fact retrieval do not only interact (as reflected by the color-changing arrow between the two anatomically separate networks in Figure 5A), but depending on the degree of difficulty of a task, the fact retrieval network and the magnitude network should be involved in all arithmetic tasks to a variable degree (Figure 5B).

In particular, in very easy tasks (not only easy multiplication but also very easy subtraction) fact retrieval is proposed to be involved independent of the operation. This is based on the assumption that more complex arithmetic problems can (and are) typically split up in subproblems, so that parts of the problem can always be retrieved as facts [139]. For instance, 74 − 52 can be split up into 70 − 50 and 4 − 2, with the latter being retrieved as fact from memory (depicted as the blue rectangle in Figure 5B cf. also [49]). However, the harder a task becomes, the less fact retrieval (see Figure 5B, blue triangle, variable fact retrieval component) and the more magnitude processing (see Figure 5B, red triangle, variable magnitude processing component) is employed. On the other hand, an invariant component of mandatorily involved magnitude processing is assumed as well, depicted as the red rectangle at the top of the figure. In sum, it is proposed that in adults with and without DD, core regions of both networks (i.e., AG and IPS) should be involved in all arithmetic tasks; the degree of activation of the two core regions should, however, be reciprocal and be dependent on overall task difficulty.

This interpretation is in line with recent data from rTMS [54]. The authors found that the involvement of IPS and left AG in subtraction and multiplication was modulated by the solution strategy employed (i.e., retrieval vs. calculation) rather than by the arithmetic operation per se (i.e., subtraction vs. multiplication). The authors suggested that solving less well-established multiplication problems was associated with procedure-based calculation and magnitude processing, while solving highly overlearned subtraction problems was associated with arithmetic fact retrieval.

Interestingly, we also found greater signal change for subtraction than multiplication in the bilateral PSPL. Activations in PSPL during arithmetic have been reported previously [41,54] and it has been suggested that these activations may indicate attentional shifts along a spatial representation of numbers, the so-called mental number line [16,17]. For example, when a Western participant calculates 15 − 8, attention might move ‘leftward’ on the mental number line from 15 to 7. Greater PSPL activation for subtraction than multiplication may reflect a more frequent use of visuo-spatial strategies during subtraction [62,140]. Alternatively, the increased PSPL activation could be related to more eye movements during subtraction. PSPL hosts the parietal eye fields [141,142,143] and more difficult tasks (such as subtraction) are typically associated with more eye movements [144]. Stimuli were presented centrally, however, our subtraction items also contained on average more digits than the multiplication items. We did not measure eye movements inside the scanner, so this alternative explanation cannot be excluded.

### 4.2. Arithmetic Networks in an Adults with Developmental Dyscalculia

The most striking differences between RM and the control group, however, were found during multiplication. RM’s behavioral performance on both arithmetic tasks in the fMRI scanner was less accurate and slower than the performance of the control participants. This was most marked for multiplication where performance differences stayed significant even after correcting for multiple comparisons. 

Based on RM’s severe difficulties with fact retrieval [48], we predicted lower AG activation during multiplication. However, contrary to our predictions, RM showed higher brain activation in the left AG and in the left IPS than controls during multiplication. This points to two possible explanations. First, the AG could have a different role during arithmetic. Previously it has been suggested that participants could bypass the use of fact retrieval in arithmetic verification tasks and use magnitude estimation as an alternative strategy [11]. Indeed, higher IPS activation, as found in RM during multiplication, has been found during magnitude estimation [145] and number comparison [19,27]. However, the use of magnitude estimation should lead to faster response times, but RM’s response times were significantly longer than those of the control group. It is more likely that the increased activation in left AG and IPS reflects the use of other bypass strategies such as procedural backup strategies like counting and effortful procedure-based calculation as well as conceptually based backup strategies like problem decomposition. While we did not record strategy use in the fMRI scanner, RM’s greater use of counting and calculation procedures to solve multiplication problems compared to fact retrieval has been well-documented [48] outside the scanner. For instance, upon being asked to solve 7 × 8, RM applied a combined retrieval and procedural strategy by first retrieving her 5-times table knowledge from memory (i.e., 5 × 8 = 40) and subsequently adding two sets of eight by counting up while using her fingers (40 + 1 + 1 + 1 + 1 + 1 + 1 + 1 + 1 = 48, followed by 48 + 1 + 1 + 1 + 1 + 1 + 1 + 1 + 1 = 56). Not surprisingly, the solution time for this problem was quite long (18.5 s). Importantly, as mentioned above, RM’s fact retrieval was restricted to rule-based facts (n + 1, n × 1) and the 5-times table. Curiously, RM could not even reliably access her 2-times table knowledge (e.g., upon solving the problem 4 × 2 she counted up by twos). Thus, it is plausible to assume that the higher activation of left and right motor cortices during multiplication in RM compared to controls suggests that she used counting-based strategies also in the scanner.

Subvocal rehearsal, as used in silent counting, activates the left inferior parietal cortex in typical adults [146,147]. Similarly, previous neuroimaging and TMS studies showed an involvement of the left IPS and the left AG during finger counting. For example, TMS over the left AG disrupted both magnitude processing and finger representations in healthy adults [148] and increased left IPS activation has been found in healthy adults in response to finger movements associated with counting compared to lip movements of number naming [149]. Thus, the greater left AG and left IPS activation shown by RM might also partially reflect her use of finger counting strategies.

Given RM’s extensive use of finger counting strategies we also expected higher activation in her left and right motor cortices than for controls during subtraction. However, there was no evidence for this for subtraction. It is possible that this is due to the nature of RM’s finger counting strategies which resulted typically in smaller finger movements during subtraction (counting down in ones) than during multiplication (counting up by ones and twos) [48]. A previous study [150] found that during complex finger movement, activation in the IPS but not in the primary motor cortex increased with the number of transitions between fingers. This might suggest that parietal areas, such as the left IPS, are more involved in keeping track of incremental changes (by one) during counting through finger discrimination [151].

The second possible explanation for higher activation in the left AG and in the left IPS in RM than in controls could be that this DD case might at least partially reflect a disconnection syndrome (e.g., [97,98,99]). Key regions such as the AG (for fact retrieval) and the IPS (for procedure-based calculation) might itself be anatomically intact but there might be aberrant white matter connectivity either between (i) these key regions or between (ii) parietal and frontal cortex. This means that (i) the connection between the fact retrieval network (Figure 5A right panel) and the magnitude processing network (Figure 5A left panel) might be disturbed, which has been suggested via U-fibers between AG and IPS (see [49]; reflected by the color-changing arrow in Figure 5A). Alternatively, (ii) connectivity between frontal and parietal areas might be reduced in RM, i.e., either in ventral pathways (e.g., via the EC/EmC system, cf. Figure 5A), in dorsal pathways (e.g., via the callosal bundle, cf. Figure 5A) or in both. However, testing this theory would require additional diffusion tractography data.

Interestingly, the adult with DD, RM, showed marginally lower signal change in the left PSPL during subtraction than controls (before correction for multiple comparisons). This could indicate that RM was using visual-spatial attentional strategies less than the control participants during subtraction. This could be related to her (marginally significantly) smaller visuo-spatial working memory (WM) capacity and is in line with theories that propose a visuo-spatial WM deficit as a core deficit in development dyscalculia.

In sum, our results suggest that during multiplication RM’s higher activation of the left AG and the left IPS reflect compensatory strategies including silent verbal counting and finger counting, while the lower signal change in parietal areas during subtraction might indicate that these problems are mainly solved by procedural strategies involving frontal regions. 

### 4.3. Limitations and Future Research

In this study we decided to focus our ROI analyses on domain-specific parietal regions (IPS, PSPL, AG) and the primary motor cortices. However, the whole-brain analyses revealed not only differences in brain activation between RM and the control group in domain-specific areas but also in domain-general frontal regions. The involvement of frontal regions in arithmetic is well-documented [21,63] and activation differences in frontal regions during arithmetic have been reported before in children with DD [103,152,153]. Furthermore, given the more frequent use of procedural strategies by RM, differences in frontal brain activations are clearly predicted. Thus, future studies should focus in more detail on atypical activation of frontal regions as well as on functional and structural connectivity between fronto-parietal cortices in adults with DD.

One of the reasons why we did not pursue this in the current paper is that RM’s strategy use is highly variable and item-dependent [48]. Thus, the expected brain activations would differ depending on the type of procedure used for each trial. This highlights the importance of obtaining a trial-by-trial report of strategy use [154] during task performance in the fMRI scanner from both adults with DD and controls in future studies. Furthermore, investigating how brain activations might be modulated by specific characteristics of the arithmetic items (such as whether the incorrect answer was table-related or unrelated, or whether the difference between the proposed and the correct answer, the so-called split, was large or small, e.g., [155]) in future studies with more items might provide additional insight into what might influence the balance of involvement of networks underlying procedure-based calculation versus those supporting arithmetic fact retrieval.

While this study highlights that single case studies of adults with DD are a starting point and can inform theory development, it is important to note that any specific individual with DD, such as RM, could of course have developed an idiosyncratic pattern of compensation that might be highly unusual and not representative of adults with DD in general. Thus, any generalization should be based on findings from much larger groups of adults with DD. In addition, our sample, both RM and the control group, consisted of highly educated university students, again limiting the generalizability of our findings.

## Figures and Tables

**Table 1:** Background variables for a single case with developmental dyscalculia (RM) and controls.

Test	RM	Controls			Significance Test ^a^		Estimated Effect Size (z *cc*) ^b^	B-H Adjusted *p*’^c^
		Mean	SD	N	*t*	*p*		
Cognitive ability (IQ) ^d^	108	121.78	10.17	18	−1.319	0.102	−1.355	0.255
Arithmetic (WRAT) ^d^	80	100.33	11.96	18	−1.654	0.058	−1.699	0.193
Spelling (WRAT) ^d^	101	111.94	4.43	18	−2.405	0.014	−2.471	0.140
Reading (WRAT) ^d^	119	113.44	6.47	18	0.836	0.207	0.859	0.259
Sight Word Reading (TOWRE) ^d^	104	103.17	9.61	18	0.084	0.467	0.086	0.467
Phonemic Decoding (TOWRE) ^d^	103	112.67	8.70	18	−1.081	0.147	−1.111	0.210
Digit Span Forward ^e^	10	12.30	1.85	10	−1.188	0.133	−1.246	0.266
Digit Span Backward ^e^	10	8.90	2.62	10	0.400	0.349	0.419	0.388
Spatial Scan Forward ^e^	7	10.90	1.92	10	−1.936	0.042	−2.030	0.210
Spatial Scan Backward ^e^	8	10.90	2.43	10	−1.139	0.142	−1.195	0.237

*Note*: WRAT = Wide Range Achievement Test. TOWRE = Test of Word Reading Efficiency. ^a^ Modified *t*-test from Crawford and Howell [105], one-tailed. ^b^ Crawford et al. [106]. ^c^ Benjamini and Hochberg correction [107] for multiple comparisons; adjusted *p*-values calculated using the method from Jafari and Ansari-Pour [108]. ^d^ Standard score. ^e^ Scaled score.

**Table 2:** Behavioral results for multiplication, subtraction and control tasks for RM and controls.

	RM	Controls		Significance Test ^a^		Estimated Effect Size (z *cc*) ^b^	B-H Adjusted *p*’^c^
		Mean	SD	*t*	*p*		
*Percentage Correct (%)*							
Multiplication	68.75	91.25	5.86	−3.737	0.0008	−3.840	0.003
Multiplication Control	93.75	97.57	2.37	−1.569	0.068	−1.612	0.090
Subtraction	57.50	78.40	10.80	−1.873	0.039	−1.924	0.078
Subtraction Control	96.25	97.01	2.92	−0.253	0.402	−0.260	0.402
*Mean Reaction Time (s)*							
Multiplication	2.815	1.63	0.30	3.867	0.0006	3.973	0.002
Multiplication Control	1.611	1.30	0.18	1.652	0.058	1.697	0.117
Subtraction	2.810	2.26	0.36	1.494	0.077	1.535	0.102
Subtraction Control	1.712	1.47	0.23	1.029	0.159	1.057	0.159

*Note*: ^a^ Modified *t*-test from Crawford and Howell [105], one-tailed. ^b^ Crawford et al. [106]. ^c^ Benjamini and Hochberg correction [107] for multiple comparisons; adjusted *p*-values calculated using the method from Jafari and Ansari-Pour [108].

**Table 3:** Brain activations by contrast for the control group.

Region ^a^	Number of voxels	Z-max	MNI Coordinates ^b^			Juelich Histological Atlas ^c^
			x	y	z	
Multiplication > Control *						
L Inferior Frontal Gyrus	3906	4.75	−46	22	20	Left Broca’s Area (45)
L Supramarginal Gyrus	1994	4.15	−44	−50	36	Left Anterior IPS (hIP1)
R Frontal Orbital cortex	1968	4.17	36	28	−6	-
L Paracingulate Gyrus	1168	3.95	−8	10	44	Left Premotor Cortex (Area 6)
R Cerebellum	487	4.17	36	−68	−46	-
L Thalamus	434	3.36	−10	−2	8	-
Subtraction > Control **						
L Inferior Frontal Gyrus	31222	6.28	−44	24	22	Left Broca’s Area (45)
L Angular Gyrus		5.95	−45	−52	54	Inferior Parietal Lobule (PFm)
R Frontal Pole		5.61	35	42	20	-
R Superior Parietal Lobule	2736	5.16	32	−64	38	Right Superior Parietal (7A)
R Occipital Pole	246	4.13	30	−90	−12	Right Visual Cortex (V3V)
Multiplication > Subtraction *						
R Precuneous Cortex	1247	4.02	10	−52	22	Right WM Cingulum
L Frontal Pole	452	4.24	−6	60	−12	-
Subtraction > Multiplication **						
L Supramarginal Gyrus	7314	5.13	−52	−42	44	Left Inferior Parietal (PF)
L Middle Frontal Gyrus	2309	4.89	−48	32	26	Left Broca’s Area (45)
R Paracingulate Gyrus	1780	5.17	6	26	36	-
R Frontal Orbital Cortex	1445	5.07	34	24	−8	-
L Insular Cortex	1201	4.65	−30	22	−6	-
R Precentral Gyrus	646	4.99	32	−4	60	Right Premotor Cortex (Area 6)
R Inferior Temporal Gyrus	375	4.18	52	−60	−14	Right Visual Cortex (V5)
R Precentral Gyrus	262	4.22	52	6	24	Right Broca’s Area (44)
R Frontal Pole	197	4.04	38	54	18	-
Brain Stem	160	3.99	0	−22	−24	-
R Middle Frontal Gyrus	145	3.98	48	30	30	Right Broca’s Area (45)

*Note*: ^a^ Regions labeled using the Harvard-Oxford Cortical and Subcortical Structural Atlases [128,129,130,131]. ^b^ MNI = Montreal Neurological Institute. ^c^ Jülich Histological (Cyto- and Myelo-architectonic) Atlas [121,122,123]. * Cluster threshold Z = 2.3. ** Cluster threshold Z = 3.1.

**Table 4:** Brain activations by contrast for the comparison of RM with the control group.

Region ^a^	Number of Voxels	Z-max	MNI Coordinates ^b^			Juelich Histological Atlas ^c^
			x	y	z	
Multiplication: RM > Control Group *						
L Precentral Gyrus	10053	4.67	−56	8	12	Left Broca’s Area (44)
L Inferior Frontal Gyrus		4.48	−52	8	16	Left Broca’s Area (44)
L Supramarginal Gyrus		3.01	−34	−38	38	Left Anterior IPS (hIP1)
L Angular Gyrus	3219	4.34	−48	−56	40	Left Inferior Parietal (PGa)
R Frontal Pole	633	4.36	24	48	24	-
R Supramarginal Gyrus	490	3.66	64	−38	40	Right Inferior Parietal (PF)
Subtraction: Control Group > RM **						
R Occipital Fusiform Gyrus	31742	5.10	16	−84	−18	-
R Thalamus		4.07	11	1	8	-
L Thalamus		4.05	−5	−10	8	-
R Middle Frontal Gyrus	1962	4.20	44	26	24	Right Broca’s Area (45)
R Frontal Pole	999	3.47	28	64	8	-
L Cerebral White Matter	776	3.74	−28	12	22	-
R Temporal Pole	590	3.44	42	22	−22	-
R Middle Frontal Gyrus	456	3.89	28	22	38	-
R Superior Frontal Gyrus	421	3.44	20	−4	66	Right Premotor Cortex (Area 6)

*Note*: ^a^ Regions labeled using the Harvard-Oxford Cortical and Subcortical Structural Atlases [128,129,130,131]. ^b^ MNI = Montreal Neurological Institute. ^c^ Jülich Histological (Cyto- and Myelo-architectonic) Atlas [121,122,123]. * Cluster threshold Z = 2.3. ** Cluster threshold Z = 3.1.

**Table 5:** Mean percentage signal change for RM and the control group during multiplication and subtraction by ROI.

Region of Interest	RM	Controls ^a^		Significance Test ^b^		Estimated Effect Size (z cc) ^c^	B-H Adjusted *p*’ ^d^
		Mean	SD	*t*	*p*		
Multiplication							
Left IPS	0.5535	0.2659	0.1488	1.881	0.0386	1.933	0.3087
Right IPS	0.1944	0.0921	0.1823	0.546	0.2960	0.561	0.3643
Left AG	0.4109	−0.0201	0.1540	2.723	0.0072	2.798	0.1157
Right AG	−0.0443	−0.1137	0.1783	0.382	0.3536	0.393	0.3771
Left PSPL	0.2913	0.1482	0.1933	0.721	0.2405	0.740	0.3498
Right PSPL	0.0965	0.0472	0.2183	0.22	0.4143	0.226	0.4143
Left Motor	0.3111	0.0920	0.1421	1.501	0.0759	1.542	0.2428
Right Motor	0.0598	−0.1134	0.1117	1.506	0.0752	1.547	0.3010
Subtraction							
Left IPS	0.2349	0.4835	0.1807	−1.339	0.0991	−1.376	0.1982
Right IPS	−0.0219	0.3397	0.2455	−1.434	0.0849	−1.473	0.2263
Left AG	−0.0374	0.1154	0.2336	−0.663	0.2580	−0.682	0.3434
Right AG	−0.2273	−0.0977	0.2418	−0.519	0.3051	−0.533	0.3487
Left PSPL	−0.1004	0.2951	0.2335	−1.647	0.0590	−1.692	0.3145
Right PSPL	−0.1602	0.2759	0.3265	−1.299	0.1056	−1.335	0.1877
Left Motor	−0.0006	0.1451	0.1584	−0.898	0.1909	−0.922	0.3055
Right Motor	−0.2928	−0.0623	0.1596	−1.409	0.0885	−1.447	0.2022

*Notes.* IPS = Intraparietal Sulcus, AG = Angular Gyrus, PSPL = Posterior Superior Parietal Lobule, Motor = Primary Motor Cortex. ^a^ N = 18. ^b^ Modified *t*-test from Crawford and Howell [105], one-tailed. ^c^ Crawford et al. [106]. ^d^ Benjamini and Hochberg correction [107] for multiple comparisons; adjusted *p* values calculated using the method from Jafari and Ansari-Pour [108].

## Data Availability

Data will be available upon request from the first author.

## Appendix A

**Table A1:** Items for the arithmetic tasks in the fMRI scanner.

Multiplication				Subtraction			
1st Operant	2nd Operant	Response	Item Type	1st Operant	2nd Operant	Response	Item Type
2	7	14	correct	11	4	7	correct
2	7	16	related	11	4	5	incorrect
7	2	14	correct	11	8	5	incorrect
7	2	15	unrelated	11	8	3	correct
2	8	16	correct	11	6	3	incorrect
2	8	15	unrelated	11	6	5	correct
8	2	16	correct	12	9	3	correct
8	2	24	related	12	9	1	incorrect
3	4	12	correct	12	5	9	incorrect
3	4	15	related	12	5	7	correct
4	3	12	correct	13	7	6	correct
4	3	10	unrelated	13	7	4	incorrect
3	6	18	correct	13	6	9	incorrect
3	6	17	unrelated	13	6	7	correct
6	3	18	correct	14	6	10	incorrect
6	3	12	related	14	6	8	correct
3	8	24	correct	14	3	9	incorrect
3	8	21	related	14	3	11	correct
8	3	24	correct	15	8	9	incorrect
8	3	22	unrelated	15	8	7	correct
3	9	27	correct	15	4	11	correct
3	9	23	unrelated	15	4	9	incorrect
9	3	27	correct	16	9	7	correct
9	3	36	related	16	9	5	incorrect
4	6	24	correct	16	3	15	incorrect
4	6	20	related	16	3	13	correct
6	4	24	correct	17	9	8	correct
6	4	22	unrelated	17	9	6	incorrect
4	7	28	correct	17	8	11	incorrect
4	7	34	unrelated	17	8	9	correct
7	4	28	correct	18	5	15	incorrect
7	4	21	related	18	5	13	correct
4	8	32	correct	18	3	17	incorrect
4	8	36	related	18	3	15	correct
8	4	32	correct	19	7	12	correct
8	4	26	unrelated	19	7	14	incorrect
4	9	36	correct	19	7	12	correct
4	9	34	unrelated	19	7	10	incorrect
9	4	36	correct	19	6	13	correct
9	4	27	related	19	6	11	incorrect
5	7	35	correct	52	39	13	correct
5	7	30	related	52	39	11	incorrect
7	5	35	correct	52	35	17	correct
7	5	31	unrelated	52	35	15	incorrect
5	8	40	correct	58	35	25	incorrect
5	8	44	unrelated	58	35	23	correct
8	5	40	correct	58	24	36	incorrect
8	5	48	related	58	24	34	correct
5	9	45	correct	64	46	18	correct
5	9	40	related	64	46	16	incorrect
9	5	45	correct	64	33	31	correct
9	5	47	unrelated	64	33	29	incorrect
6	7	42	correct	69	47	24	incorrect
6	7	44	unrelated	69	47	22	correct
7	6	42	correct	69	36	33	correct
7	6	35	related	69	36	35	incorrect
6	8	48	correct	73	57	18	incorrect
6	8	42	related	73	57	16	correct
8	6	48	correct	73	46	27	correct
8	6	52	unrelated	73	46	25	incorrect
7	8	56	correct	75	48	29	incorrect
7	8	58	unrelated	75	48	27	correct
8	7	56	correct	75	34	39	incorrect
8	7	48	related	75	34	41	correct
7	9	63	correct	86	59	27	correct
7	9	56	related	86	59	25	incorrect
9	7	63	correct	86	43	45	incorrect
9	7	61	unrelated	86	43	43	correct
8	9	72	correct	87	69	18	correct
8	9	76	unrelated	87	69	16	incorrect
9	8	72	correct	87	58	31	incorrect
9	8	81	related	87	58	29	correct
3	7	21	correct	91	38	53	correct
3	7	24	related	91	38	51	incorrect
7	3	21	correct	91	76	17	incorrect
7	3	19	unrelated	91	76	15	correct
2	9	18	correct	95	21	76	incorrect
2	9	21	unrelated	95	21	74	correct
9	2	18	correct	95	28	67	correct
9	2	16	related	95	28	65	incorrect
